# Is the capacity to consent different from the capacity to refuse treatments and procedures in adolescence?

**DOI:** 10.1016/j.jped.2025.04.004

**Published:** 2025-05-15

**Authors:** Guilherme Henrique Martins, Kalline Eler, Aline Albuquerque, Rui Nunes

**Affiliations:** a Faculty of Medicine, University of Porto, Porto, Portugal; b Federal University of Juiz de Fora, Juiz de Fora, MG, Brazil; c University of Brasilia, Brasília, DF, Brazil

**Keywords:** Decision-making, Adolescent, Bioethics, Clinical ethics

## Abstract

**Objective:**

The objective of this article is to broaden the discussion on the factors that constitute adolescent healthcare decisional capacity, ensuring that adolescents are recognized as capable of refusing treatments or procedures.

**Sources:**

Materials from different sources were analyzed, including articles from reputable databases and documents from government agencies, forming a purposefully selected sample. The research was conducted in two phases: document selection and reflective analysis, followed by a report. The discussion was approached from a phenomenological perspective, with reflections grounded in human rights principles.

**Summary of the findings:**

Healthcare decisional capacity must be sufficiently robust to allow adolescents to refuse treatments or procedures. It is essential to respect the right of capable adolescents to refuse treatments and procedures. Protecting the vulnerability of adolescent patients involves honoring their growing autonomy. Data from field research regarding the refusal of treatments and procedures in adolescence are scarce, which limits the scope of the proposed discussion.

**Conclusions:**

It cannot be argued that adolescents should have different abilities to refuse a treatment or procedure compared to those required to give consent. The importance of these skills seems to vary between these situations. This difference is justified by the need to consider potential harm to health, even though it could be argued that damage to health should be part of the bioethical deliberation surrounding the decision, rather than a factor in the assessment of decisional capacity.

## Introduction

Decision-making in clinical contexts is one of the most complex aspects of the relationship between healthcare professionals and adolescent patients, involving the expectations and perspectives of all parties. This complexity arises from the wide range of intrinsic and extrinsic factors that influence the patient's decision-making capacity, as well as considerations related to damage to health, potential benefits, and alternatives to the proposed diagnostic or therapeutic approach.^1^ In this context, healthcare professionals must adopt behaviors that align with the provision of quality care and respect for the patient's rights. This includes obtaining the patient's consent for a given proposal and considering their will, preferences, and sense of well-being within the framework of Shared Decision Making.^2^^,^^3^ Clinical decisions during adolescence are particularly significant in this scenario especially when the patient exercises their human right to refuse treatments and procedures.^4^^,^^5^

From a human rights perspective, adults, children, and adolescents are all entitled to the same fundamental rights, as they share the status of human beings.^6^ In this context, the right to refuse treatment or procedures, considering the human right to privacy, is universally recognized and has been a central topic in academic discussions since the 1980s.^7^ Concerning children, the entitlement to human rights is grounded in a robust theoretical-normative framework, particularly the Convention on the Rights of the Child (CRC), adopted by the United Nations in 1989.^8^ Article 16 explicitly guarantees the right to privacy, prohibiting arbitrary or unlawful interference in a child's private life.^4^^,^^8^ However, despite this strong normative foundation, the right of children and adolescents to refuse remains underexplored in the specialized literature, particularly in the context of healthcare issues.^9^^,^^10^ The main issues in this area involve balancing the respect for privacy, which includes the right to consent or refuse, with the duty to safeguard health. This balance also entails the involvement of legal guardians and healthcare professionals, alongside the need for evidence-based assessments of healthcare decisional capacity.^3^^,^^4^ In some European countries, including the United Kingdom and Portugal, individuals aged sixteen and over are generally presumed capable of making healthcare decisions.^11^^,^^12^ However, in the United Kingdom, even when an adolescent refuses medical treatment, his or her legal guardians can give consent on his or her behalf. This not only creates a bioethical problem regarding the limits of parental authority but also raises concerns about legal uncertainty.^10^^,^^13^^,^^14^ In Latin American countries, such as Brazil, the situation is even more complex, requiring legislative advances to guarantee the right to respect for patients’ private lives. In Brazil, the law generally considers individuals under the age of eighteen legally incapable of making autonomous decisions regarding personal matters, including their healthcare.^3^^,^^15^

It is clear that, from the perspective of Clinical Bioethics grounded in the theoretical framework of human rights as applied to healthcare, all adults are presumed capable of making decisions regarding their lives, including health-related matters, based on their will and preferences.^3^ When it comes to adolescents, their decision-making capacity evolves, as acknowledged in Article 5 of the Convention on the Rights of the Child (CRC), which acknowledges their evolving capacities.^8^ This implies that their ability to make autonomous health-related decisions may fluctuate throughout adolescence. When this capacity is in question, it should be assessed based on scientific evidence. In this context, it is recognized that the more an adolescent learns, experiences, and understands, the more the healthcare team and legal guardians can shift from guiding, offering reminders and advice, and eventually fostering an exchange of knowledge and opinions on equal terms.^16^ Along these lines, the *heightened vulnerability* of adolescent patients is also acknowledged. Although all individuals are inherently vulnerable, which implies a universal and indiscriminate susceptibility to physical or psychological harm, child and adolescent patients face heightened vulnerability. This is because adolescents have limited resources to prevent health damage from occurring or to respond effectively to it, compared to adults. These limited resources include, for example, a set of factors intrinsic to adolescent development, which have already been analyzed by specialized literature.^1^^,^^6^^,^^17^^,^^18^ Consequently, decision-making in adolescents should be guided by approaches that acknowledge their evolving capacities while providing professional support proportional to their vulnerabilities.

*Adolescent healthcare decisional capacity* refers to the ability of adolescent patients to make health-related decisions based on their own will and preferences. Its assessment is a crucial requirement for obtaining informed consent in healthcare and, consequently, for exercising the patient's human rights.^3^^,^^19^ Given that this capacity is intertwined with everyday health decisions in adolescents, who present unique complexities, this article will not specifically address topics such as permanent or temporary sterilization, pregnancy, sexual health and gender-related issues, aesthetic procedures and treatments for cosmetic purposes, treatments for eating disorders, experimental therapies, palliative care, or end-of-life care. Furthermore, this theoretical article explores decision-making capacity regarding consent and refusal, based on the premise that, in comparison to the capacity to consent, a more robust capacity should be required for an adolescent to refuse a treatment or procedure, given the potential risks of harm associated with such a decision.^11^ Thus, the idea is supported that the greater the risk of damage to health, the greater the level of understanding an adolescent patient must have to make an informed decision. Consequently, the primary concern in assessing this capacity should be determining whether the adolescent is sufficiently capable, at a given time, to either consent to or refuse a treatment or procedure.^1^ Although this is a complex issue, the authors have chosen to expand the discussion due to the need to ensure that the rights of this specific population are not compromised by paternalistic and outdated healthcare practices. The reflection proposed in this study addresses the assessment of adolescent healthcare decisional capacity when the adolescent exercises their right to refuse treatments and procedures. It considers the fact that the greater the potential harm, the more *robust* the skills that constitute decisional capacity must be, and therefore, the more precise its evaluation should be.^6^^,^^20^, ^21^, ^22^ The authors use the term 'robust' to signify that decision-making skills involved in refusing treatment require greater strength and depth, due to the potential consequences of such a decision. This demands a more firm and stable understanding from the patient regarding the implications of their choice.

In the context of an adolescent patient's refusal of treatments and procedures, the care team often faces the following question: Should the adolescent's refusal be respected? This article proposes a new approach by asking: *Does adolescent healthcare decisional capacity entail sufficiently robust skills for the adolescent patient’s refusal of treatments and procedures to be respected?* While many studies discuss and apply fundamental concepts to the decision-making process in terms of the capacity to consent, few address the ability to refuse treatments and procedures.^23^ From this perspective, there is a gap in the specialized literature regarding the bioethical aspects of adolescent healthcare, which this article aims to contribute. It seeks to reflect on a topic that has not yet been adequately explored. Therefore, the article’s objective is to contribute to the ongoing discussion of the elements that constitute adolescent healthcare decisional capacity, ensuring that the patient's right to refuse treatments and procedures is effectively upheld in clinical practice.

## Methods

This article is the result of a theoretical, documentary study.^24^^,^^25^ It is grounded in the framework of Clinical Bioethics as outlined by *Healthcare Bioethics*, which is structured on three pillars: Patient-Centered Care, Shared Decision-Making, and Patients’ Rights.^26^ Additionally, it is based on the bioethical principles established by the CRC and the guidelines on autonomy and *decisional capacity* proposed by Eler and Albuquerque.^19^^,^^22^ Two key reasons are provided for the selection of the term *capacity* in this study: (1) *Competence* is a legal term primarily employed within the judicial system, whereas *capacity* is more frequently used by healthcare professionals. (2) in its specific context, the term *competence* also encompasses an individual’s ability to make decisions on a range of life matters, not limited to healthcare-related issues.^27^^,^^28^ Additionally, *capacity* is more commonly referenced when discussing decision-making abilities from a human rights perspective, as seen in the General Comments of the Committee on the Rights of Persons with Disabilities.^29^ This ability is also referred to as *health capacity or health competence or mental capacity, or mental competence*, depending on the legal and bioethical traditions of the country in which the term is used.^22^ Similarly, although the CRC does not provide an accurate definition of adolescence or adolescence, later documents indirectly address this concept – namely, General Comment No 20, which focuses on the implementation of the rights of the child during adolescence, categorizes this age group as ranging from ten to eighteen, thereby defining the age span covered by this article.^30^

To address this topic about this audience, the analysis included a review of books, academic journal articles, and white papers of relevance, particularly those indexed in databases with robust bibliometric metrics. Additionally, the websites of international institutions and their affiliated agencies were checked, along with guidelines provided by reputable governmental and non-governmental organizations in the field of safe healthcare. A theoretical intentional sampling was applied to the entire set of documents reviewed.^24^ Saturation in the document search was reached when researchers no longer found new insights after multiple cycles of data collection.^31^ Documents were selected based on four scientific criteria: authenticity, credibility, representativeness, and significance.^32^ The research was conducted in two stages, resulting in the preparation of a report, which included: document selection and reflective thematic analysis.^25^ The reflective thematic analysis was employed to incorporate the researcher’s subjectivity as a valuable scientific resource when interpreting concepts and phenomena, particularly through the lenses of bioethics and human rights.^25^

A phenomenological epistemological stance was adopted for analyzing the documents and their contents, allowing for both objective and subjective interpretations.^32^ A critical perspective was taken, grounded in the understanding that bioethical challenges are shaped by diverse sociocultural contexts. Consequently, human rights must play a role in addressing and resolving these issues.^33^ Regarding the understanding of adolescent healthcare decisional capacity specifically, the Human Rights Model framework was chosen over the Gillick Competence Theory, originating in the United Kingdom, and the Mature Minor Theory, of American origin. These models are now considered insufficient for application in the healthcare context.^34^, ^35^, ^36^, ^37^ The selection of the Human Rights Model was based on its emphasis on a fundamental principle for the authors: ensuring the adolescent's right to actively participate in their healthcare.^22^

To minimize the impact of potential biases in the studies supporting this research and to generate original knowledge, information collected from different research methods was analyzed. This included both primary data, gathered through qualitative and quantitative approaches, and secondary data from theoretical research and literature reviews. This approach enabled data triangulation, fostering the generation of empirical, inductive knowledge and ensuring that the data discussed were not derived from a single scientific perspective.^24^ Only studies with well-defined research practices, aligned with scientific integrity standards, were included.^38^

### Aspects of the assessment of adolescent healthcare decisional capacity

*Healthcare decisional capacity* involves four essential components of patient skills, widely recognized and collectively known as the *four-skills model*: understanding, appreciation, reasoning, and expression of choice.^39^ In applying this model, for an adolescent patient suspected of having an undiagnosed heart condition to consent to a diagnostic procedure that involves risks, they must understand the proposed procedure, assess its potential consequences, process the information rationally, and, as far as possible, express their decision. Assessing these skills is crucial in the context of adolescent healthcare, as it provides a necessary foundation for discussions about their rights, including the right to consent to or refuse medical procedures and treatments.^6^ However, the *four-skills model* is not without its criticisms and is considered by some to be insufficient, particularly when it overlooks factors such as the impact of the individual's decision during the capacity assessment, favoring a more “cognitive” approach to decision-making.^40^, ^41^, ^42^ In this area, concerns aligned with the authenticity of decision-making also deserve to be highlighted, even if under the aegis of arguments with which the authors disagree.^43^ Nevertheless, since the pioneering work of Appelbaum and Grisso,^39^ other studies have gradually contributed to the field, largely in alignment with the framework established by these authors.^1^^,^^28^^,^^44^, ^45^, ^46^, ^47^, ^48^, ^49^, ^50^, ^51^, ^52^, ^53^, ^54^, ^55^, ^56^

Given the above, assessing the decision-making capacity of adolescent patients requires a professional approach that considers multiple factors, extending beyond analyses that focus just on age or cognitive development.^1^ This perspective acknowledges adolescents as rights-holders, whose will and preferences should not only be heard but also respected, to the extent that they can make informed decisions. Additionally, several other points must be clarified in light of scientific evidence:

1. All decisions involving adolescent patients must provide them with the opportunity to actively participate, allowing them to express their feelings, opinions, concerns, fears, values, and preferences.^57^ This is not only a matter of their inherent rights but also reflects the interest of adolescent patients in decisions about their health, which range from talking about contraception to discussions about end-of-life care in the context of serious illness.^23^^,^^58^^,^^59^ When capable of making decisions, the adolescent's will and preferences must be respected, except in very specific circumstances where damage to health appears to be a theoretical-practical framework that will be discussed below. When unable to make decisions, so-called decision-making support mechanisms must be considered to promote their autonomy. Only then should substituted decision-making mechanisms be triggered.^3^
2. Adolescents between the ages of fourteen and fifteen may be capable of making decisions comparable to those of adults, a fact that is not entirely new.^60^ This means that within this age range, adolescents can make informed decisions about their health care, even in diverse clinical settings.^61^ This understanding is not contrary to, but rather a perspective that complements, the *maturity gap* typical of adolescents, a period in which the cognitive component reaches acceptable levels around the age of sixteen, while psychosocial maturity continues to develop.^62^ It is important to emphasize that the aim is not to use age as an isolated criterion, but as part of a broader set of determinants, reflecting the understanding that age alone does not justify denying the patient's right to consent, participate in, or refuse health decisions.
3. The assessment of adolescent healthcare decisional capacity, when aligned with patient rights, cannot rely just on cognitive approaches.^40^, ^41^, ^42^^,^^63^ It is essential to demystify the idea that decision-making capacity is only proportional to the isolated cognitive abilities of the adolescent patient.
4. The assessment of adolescent healthcare decisional capacity must consider both intrinsic factors (such as age, gender, cognitive and pubertal development, general and specific medical history, maturity, and life experiences) and extrinsic factors (including family support, healthcare professional support, and environmental conditions like culture, housing, and social relationships).^1^ This perspective arises from the understanding that adolescents are not isolated individuals, detached from the influences of their surroundings and the people around them.^7^ Furthermore, adolescents make decisions within a specific context, and this must be considered when assessing their healthcare decisional capacity.

The specialized literature has focused on developing technical tools to assess healthcare decisional capacity, primarily concentrating on adults.^64^ When it comes to assessing the same capacity in adolescents, most scientific research focuses on instruments that facilitate Shared Decision-Making, rather than focusing specifically on assessing decision-making capacity.^65^ An exception to this trend is the *MacArthur Competence Assessment Tool for Treatment (MacCAT-T)*, the most well-known instrument for assessing consent to clinical treatment in adults, which has been considered viable for adaptation to adolescents.^66^ In this way, the *Children's Competence in Decision-Making (CCDM)* was developed, tested, and validated, a scale designed to measure the decision-making capacity in health care of children aged eight to twelve years with bronchial asthma and type 1 diabetes mellitus.^67^ Similarly, the *MaturTest* in Spain, a reasoning test focused on moral conflicts among adolescents, based on the levels and stages of moral.^68^^,^^69^ Interestingly, in a subsequent study, the same team compared the results of the instrument with the maturity of patients as subjectively assessed by parents and pediatricians but found no correlation.^70^ Additionally, it was a study using the *Melbourne Decision-Making Questionnaire (MDMQ)*, validated through a cross-sectional study (*n* = 822) involving adolescents aged fourteen to eighteen in Colombia.^71^ They proposed categorizing adolescent patients based on their decision-making styles into four categories: vigilance, hypervigilance, buck-passing, and procrastination. To address the issue, the WHO published guidelines outlining a four-step approach to assess and support adolescents' capacity for autonomous decision-making: joint exploration of situations and options, joint synthesis of the situation, decision-making point, and follow-up.^16^^,^^72^ However, it is concerning that the difficulty of cross-cultural adaptation and the statistical reliability of the psychometric scales used in these instruments are two key factors contributing to the scarcity of these important tools.^41^

The primary goal of these technical instruments is to assess whether, at a given moment and in a specific situation, the adolescent patient is capable of making decisions independently, that is, whether they are capable of exercising their right to privacy, which includes the right to make decisions. It is important to recognize that the status of incapacity should not be assigned to an adolescent who possesses decision-making capacity. Therefore, it would also be a mistake to assume that adolescents are more capable and self-sufficient than they are, necessitating a thorough analysis of their abilities by healthcare professionals.^6^ On the other hand, an eminently paternalistic stance might argue that, due to their vulnerability, adolescents should be protected from the risk of harm. However, this perspective needs to be very carefully considered.^6^ In the context of Clinical Bioethics grounded in human rights, it has been emphasized that when adolescents are capable, therefore, if they understand the risks, benefits, and alternatives presented by the care team and can weigh the potential damage to their health, they should, as a general rule, have their right to refuse treatments and procedures respected, as will be discussed further below.

### The adolescent healthcare decisional capacity to refuse treatments and procedures

Recent discussions on the refusal of treatments and procedures in adolescence encompass a range of theoretical and practical elements, with a particular focus on patient rights protection and empowering adolescents to make their own decisions.^73^ Respect for the adolescent patient's right to refuse treatments and procedures is especially prominent in this context due to its connection with the assessment of the patient's decision-making capacity, as it is essential to determine whether the adolescent is deemed capable of making decisions, including the specific decision to refuse treatment.^6^ The main focus of the points discussed here is to deepen the conversation about assessing healthcare decisional capacity in situations where adolescent patients refuse treatments or procedures. The goal is to argue that this assessment should account for more robust skills as the severity of potential consequences, risks, and uncertainties of the proposed diagnostic or therapeutic interventions increases, taking into consideration the urgency (or lack thereof) of the health decision at hand.^11^^,^^74^, ^75^, ^76^ This proposition extends a principle regarding consent, rather than refusal, as follows: an adolescent who is capable of consenting to a relatively low-risk treatment may not necessarily have the capacity to consent to a more complex treatment involving higher risks or serious consequences.^11^ In the case of the capable adult, on the contrary, the proportionality of risk and harm are not central to the bioethical deliberation of their decision, which must inevitably be respected. Therefore, the potential risk of *damage to health* becomes a crucial factor in decision-making when the adolescent exercises their right to refuse. However, it is important to emphasize that this factor does not, by itself, serve as a determinant for *assessing* capacity. Instead, it is a key element in the *bioethical deliberation* regarding whether to accept or override their decision, a complex issue beyond the scope of this article.

The World Health Organization defines *harm to health* as any impairment of structure or function of the body and/or any deleterious effect arising there from disease, injury, suffering, disability, and death.^77^^,^^78^ Similarly, the General Medical Council (GMC), the public body responsible for regulating the medical profession in the United Kingdom, broadens the concept to encompass any potential negative outcome resulting from a healthcare intervention, including side effects or complications, referring to it as "damage to health".^11^ Both the expressions *damage to health* and *harm to health* are used in the literature to refer to the same concept. For the present discussion, these terms are considered synonymous and are employed interchangeably to address the terminological issue. The existing research on the relationship between the right to refuse treatments and procedures, damage to health, and healthcare decisional capacity among adolescent patients is, generally limited, with some findings being relatively old or even outdated. Nonetheless, these studies remain significant.

The bibliographic mapping highlights three key points, which will now be emphasized: (1) The adolescent's decision-making capacity regarding the refusal of treatments and procedures must be evaluated using rigorous criteria to accurately assess their decision-making ability. This should involve scientifically validated tests, particularly when the decisions carry higher risks.^27^^,^^35^^,^^79^ (2) Respecting the right of capable adolescent patients to refuse treatment appears to be the most consistent approach to honoring their human rights. Particularly in situations involving serious risks to physical or mental integrity, where potentially irreversible damage to health may occur, there is no consensus on the extent of judicial, professional, or family intervention in the adolescent's decision. However, damage to health serves as a relevant criterion to guide bioethical discussions on the matter.^11^^,^^20^^,^^22^ (3) It is essential to protect adolescents from making decisions that could lead to significant harm, while also considering their evolving decision-making capacities. This balanced approach aims to prevent holding them accountable for responsibilities that exceed their capacity while ensuring the respect of their human rights.^6^^,^^10^^,^^80^ To facilitate the following discussions, the relevant findings are organized in Table 1.^81^, ^82^, ^83^

**Table 1:** Current understanding of the relationship between the right to refuse treatments and procedures, harm to health, and healthcare decisional capacity in adolescent patients.

References	Consideration
Buchanan and Brock^78^	For the child [and adolescent] to achieve the right to refuse, the capacity must be greater than the capacity to consent.
Pearce^79^	A more rigorous assessment should be applied when evaluating a child's ability to refuse consent compared to assessing their competence to give consent.
Doyal and Henning^80^	A competent adolescent has the moral right to decide whether to continue treatment or to cease it, especially in cases of chronic illness and terminal conditions.
Shaw^13^	The level of understanding required for an adolescent to make a decision is directly proportional to the risk-benefit ratio of the proposed treatment.
Stancioli^81^	Two criteria must be considered before decision-making power is granted to a teenager: preventing the legal system from becoming excessively complex and evaluating the risks associated with the decision-making process.
Annas^20^	The right to refuse should prevail in the decision when there is no risk of death or severe harm to the patient's health.
Cave^35^	The more severe the potential outcome, the higher the standard of proof [that assesses decisional capacity]
Michaud et al.^27^	The adolescent patient's evolutionary capacity to make decisions is proportional to the complexity of the decision.
Manson^82^	Consent and refusal have normative power with respect to adolescents, but refusals are limited by situations where serious harm may occur.
Kling & Kruger^83^	Medical treatment should only be provided or withheld if the patient has given legal consent or refusal.
GMC^11^	The harm to adolescents' rights must be carefully considered when overriding their refusal, ensuring decisions are made in their best interests.
Eler^22^	Disregarding the adolescent's expressed wishes is only possible if the risks associated with their choice prove to be contrary to their best interests.
Skelton, Forsberg & Black^10^	It is important to protect adolescents from full responsibility for their decisions, which may mean that refusals associated with harm to health may not be normatively decisive.
Herring^6^	Harm must be prevented at all costs by the care team and legal guardians of adolescent patients.

Source: own authors.

The National Council of Ethics for Life Sciences (CNECV), an entity linked to the Portuguese constitutional public administration, has shared its position on the informed consent process for adolescents, offering valuable contributions. The council has categorized decisions into two types based on the potential harm they may cause: *minor acts* (referring to decisions that do not jeopardize the adolescent's life) and *major acts* (referring to decisions that involve risks to the adolescent's life or integrity or have a significant impact on their life).^12^ Although this categorization is applied to situations where there is disagreement between the adolescent's legal representatives regarding parental responsibilities, rather than cases where there is doubt about the adolescent healthcare decisional capacity, it still underscores the importance of understanding damage to health as central to discussions on decision-making in adolescence. This perspective introduces a framework for evaluating the consequences of damage to health, which appears logical when classifying it into two categories: *(a) life-threatening or with serious health repercussions, and (b) non-life-threatening or without significant health consequences.* Thus, it would be reasonable to accept a decision made by the adolescent, even if deemed inappropriate from the perspective of the healthcare professional, as long as it does not result in a risk to the patient’s life or cause serious harm to their health. The effort to outline such practical guidelines is commendable, especially when considering the daily bioethical challenges that healthcare professionals must address promptly. However, despite recognizing this, the authors believe that it is not feasible to generalize the issue in such a manner. The need to establish exceptions to this understanding, particularly in situations such as palliative care and/or end-of-life scenarios, highlights the limitations of categorizing refusal of treatments and procedures in adolescence in this manner. Therefore, there is a risk that such generalization becomes inadequate, as it may fail to account for the specificities of each case, whether regarding the adolescent's decisional capacity or the decision itself, even if that decision increases the risk of harm, it may, from a holistic perspective, ensure their well-being and quality of life. Thus, it is suggested that the concept of damage to health be addressed only after the assessment of decision-making capacity, which should be conducted on a case-by-case basis, considering the unique circumstances and timing of each situation.^56^

The susceptibility of adolescent patients to refuse treatments and procedures exists in certain situations for various reasons, and denying this fact is incompatible with providing safe and effective healthcare. Therefore, contrary to what might initially be argued, this discussion does not advocate for adolescent patients to exercise autonomy they are not capable of, but rather seeks to challenge the notion of presumed incapacity that is upheld by professional practice, paternalism, and forced beneficence. Advocating for a more restricted view of autonomy in adolescence, bioethical literature grounded in Principlism argues that religious beliefs, for instance, may not remain consistent throughout one's life, thus, such beliefs could not justify a refusal in this age group, given the "state of dynamic identity flux" that makes decision-making capabilities in adolescents more uncertain.^43^ In theory, this situation could lead to damage to health that is too significant to be ignored, thereby necessitating a justified limitation of the right to refuse, regardless of the patient's capacity.^43^ However, analyzing the issue from such a categorical perspective presents several challenges. First, it fails to consider the adolescent patient's evolving capacity, and second, the idea of a "dynamic state of identity flux" cannot, on its own, justify denying the right to refuse, especially when well-established Shared Decision-Making mechanisms exist to support the adolescent's autonomy.^3^ The refusal of treatments and procedures should not be analyzed solely through the lens of typical physiological development during adolescence, as doing so risks stigmatizing this age group.^84^ The discussion surrounding an adolescent patient's right to refuse should not require decision-making skills that differ from those necessary for consenting to treatment in this age group. Rather, it should emphasize that, alongside these rights, the adolescent's ability to understand the potential irreversibility of the consequences must be considered, as well as the possibility of any future regret regarding the decision made.^85^

In discussions on this topic, some authors have supported a bioethical approach based on moral philosophy, where a patient's ability to refuse treatment or procedures directly depends on the severity of the situation requiring that the adolescent not only understand the decision at hand but also undergoes a subjective validation of its rationality by the health professional.^86^, ^87^, ^88^, ^89^, ^90^, ^91^ This perspective outlines the concept of *Drane Competence*, which is limited by the challenge of determining whether an adolescent is capable of making decisions until they either accept or refuse the proposed care. In other words, it ties capacity to the outcome of the patient's decision, rather than evaluating the skills they possess before making a choice.^85^ Additionally, models that treat damage to health as a key determinant in assessing decision-making capacity have faced criticism in the literature, since, in this aspect, the assessment of the decision would be seen from the health professional's perspective rather than being centered on the patient themselves.^85^ Although open to debate in terms of the role of professionals in determining decisional capacity, this perspective does not diverge when it comes to the necessary skills required to refuse treatment or procedures, in contrast to those needed for consent, as outlined in the previous section of this article.

Although these are specific cases, and therefore limited in terms of scientific generalization, the specialized literature provides valuable reports on adolescent refusals that offer important perspectives. Notably, these include an outdated discussion about parental autonomy in deciding for capable adolescents and a pioneering account of the adolescent’s self-awareness regarding the need for a deeper understanding of their abilities to refuse a treatment or procedure.^14^^,^^63^^,^^84^, ^85^, ^86^^,^^92^, ^93^, ^94^, ^95^ Given this, it seems reasonable to argue that the authors should aim for a more clearly defined understanding of the skills that constitute an adolescent's decision-making capacity in healthcare, referred to as robustness in this article, specifically in situations involving the refusal of treatments and procedures, as listed in a specific section. In this case, for the adolescent patient's will and preferences to be adequately balanced with their protection, the literature lists some conditions, namely: (1) the impacts of the decision must be identified and understood by all involved, (2) the main objective of the professional approach must be to facilitate a broad and voluntary decision and (3) the eventual failure to facilitate a broad and voluntary decision within the relevant timeframe must lead to a decision that coincides with the adolescent's best interests.^96^ It is crucial that all parties involved in the decision-making process fully understand the implications of the decisions made by an adolescent, ensuring an informed and conscious choice. The professional’s role is to support the adolescent in making an autonomous decision, creating an environment that respects their right to make choices about their own life. In situations where this is not feasible, such as in urgent or emergency cases, the professional's intervention must ensure a decision is made that prioritizes the adolescent's best interests, focusing on their protection and well-being.

**Figure 1:**
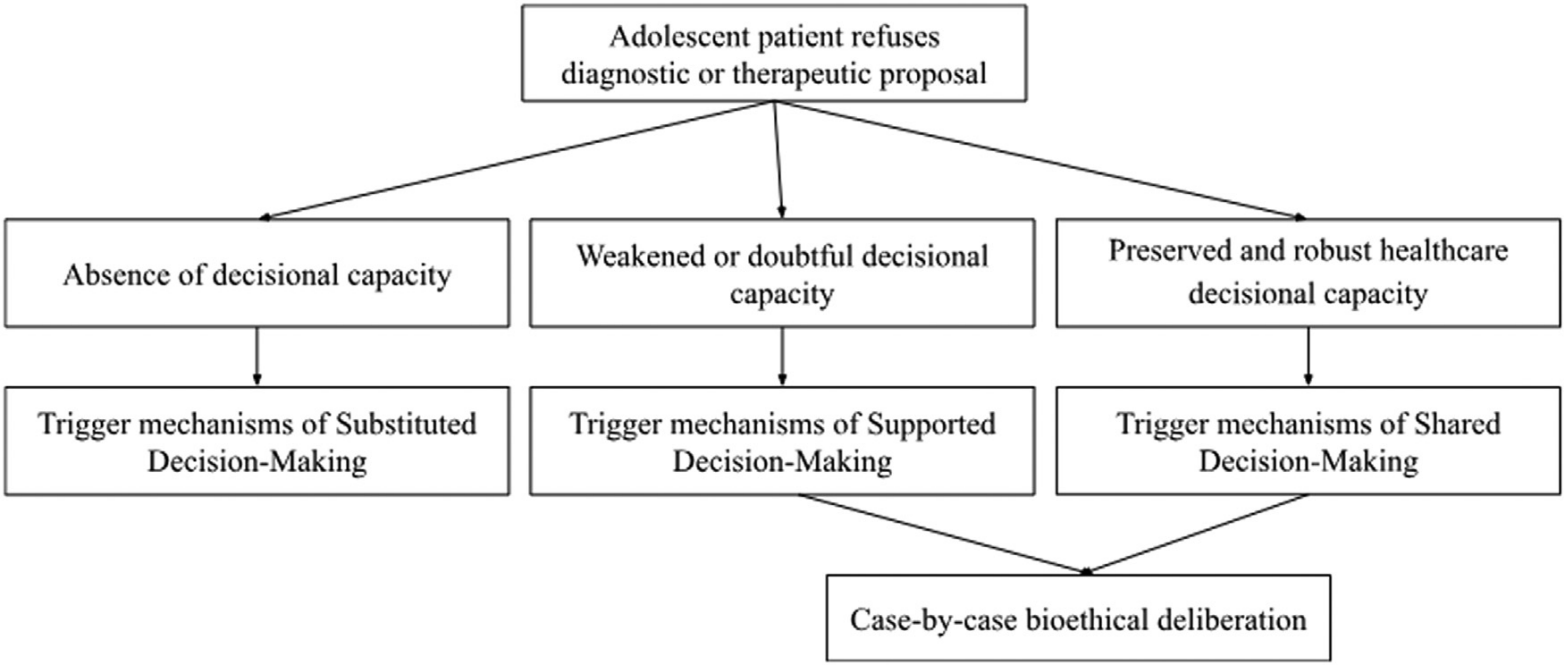
Proposal for bioethical deliberation upon refusal of diagnostic or therapeutic proposal in adolescent patients in the context of clinical care. Source: Own authors.

It is important to clarify that while the literature often supports the need for a standardized step-by-step guideline regarding the refusal of treatments and procedures for adolescents, the authors have deliberately chosen not to pursue that approach. While such a guideline may be desirable in daily clinical practice, creating a bioethical prescription at this stage could oversimplify the complexity of the issue and overlook the theoretical and practical gaps that still exist in both the literature and scientific studies. This would, in our view, be imprudent. However, a path is proposed, based on the need to maintain a balance between the right to refuse treatments and procedures by adolescents and the right to protection of these patients due to their increased vulnerability, taking into account the right to be heard, not to be discriminated against, the principle of best interests and the right to adult support to be respected.^97^ This bioethical deliberation, which is not the subject of study in this article, is offered as a guiding framework for future discussions, as illustrated in Figure 1. Furthermore, other mechanisms should be considered in adolescent decision-making to promote the autonomy of this patient and can be found in the specialized literature.^19^

A theoretical model that validates the right to refuse treatments and procedures, based on the identification of healthcare decisional capacity and considering the damage to health, is not without its criticisms.^44^^,^^97^, ^98^, ^99^, ^100^, ^101^, ^102^ Although these criticisms are becoming less frequent, they exist and often reflect a paternalistic bias. For instance, it is suggested that an individual may be deemed capable of refusing treatment, but not capable of actually carrying out such a refusal when faced with a diagnostic or therapeutic proposal.^82^ This work, in contrast, argues that when an adolescent possesses the capacity to make decisions on a given matter at a particular moment in their life, their will and preferences must be respected. However, it is important to emphasize that the decision-making capacity of an adolescent in situations involving the refusal of treatments and procedures should be based on more robust skills than those required for consent.

## Final remarks

Discussing healthcare decisional capacity in complex and controversial situations challenges the weight the authors place on patients' human rights, especially the patient's human right to respect for private life. Determining adolescent healthcare decisional capacity in cases where treatments or procedures are refused helps reduce the paternalistic imposition of health professionals' perspectives on adolescent patients. In this sense, it cannot be asserted that adolescent patients should be required to have different skills to refuse a treatment or procedure compared to those needed to provide consent. However, it is important to note that the robustness of these skills seems to differ between situations, being more solid and profound in the case of refusal, considering the potential damage to health arising from the decision. This does not serve as a justification for limiting the right to refuse treatments and procedures, even when the situation is complex and the consequences are difficult to measure. A case-by-case analysis, grounded in bioethical principles based on human rights, represents a practice that aligns with the respect for privacy and the promotion of safe and effective healthcare. Despite the theoretical and practical conclusions drawn above, uncertainties remain regarding purely practical aspects in specific clinical situations, making field research highly encouraged.

## Authors contributions

Guilherme Henrique Martins: Conceived and designed the study, performed the acquisition, analysis and interpretation of data and final approval of the submitted version. Kalline Eler: Conceived and designed the study, critically revised it for intellectual content and final approval of the submitted version. Aline Albuquerque: Conceived and designed the study, critically revised it for intellectual content and final approval of the submitted version. Rui Nunes: Performed critical review of the intellectual content and final approval of the submitted version.

## Conflicts of interest

The authors declare no conflicts of interest.
